# Cuproptosis engages in c-Myc-mediated breast cancer stemness

**DOI:** 10.1186/s12967-023-04204-5

**Published:** 2023-06-23

**Authors:** Runtian Wang, Kun Xu, Qin Chen, Qin Hu, Jian Zhang, Xiaoxiang Guan

**Affiliations:** 1 Department of Oncology, The First Affiliated Hospital of Nanjing Medical University, 300 Guangzhou Road, Nanjing, 210029 China; 2 Department of Cardiothoracic Surgery, Affiliated Hospital of Nantong University, Medical School of Nantong University, Nantong, 226001 China; 3 Phase I Clinical Trial Center, Fudan University Shanghai Cancer Center, Shanghai, China; 4 Jiangsu Key Lab of Cancer Biomarkers, Prevention and Treatment, Collaborative Innovation Center for Personalized Cancer Medicine, Nanjing Medical University, Nanjing, 211166 China

**Keywords:** Intra-tumoral heterogeneity, c-Myc, Cancer stem cell, Copper-induced cell death, Cuproptosis, Tumor microenvironment

## Abstract

**Background:**

Intra-tumoral heterogeneity (ITH) is a distinguished hallmark of cancer, and cancer stem cells (CSCs) contribute to this malignant characteristic. Therefore, it is of great significance to investigate and even target the regulatory factors driving intra-tumoral stemness. c-Myc is a vital oncogene frequently overexpressed or amplified in various cancer types, including breast cancer. Our previous study indicated its potential association with breast cancer stem cell (BCSC) biomarkers.

**Methods:**

In this research, we performed immunohistochemical (IHC) staining on sixty breast cancer surgical specimens for c-Myc, CD44, CD24, CD133 and ALDH1A1. Then, we analyzed transcriptomic atlas of 1533 patients with breast cancer from public database.

**Results:**

IHC staining indicated the positive correlation between c-Myc and BCSC phenotype. Then, we used bioinformatic analysis to interrogate transcriptomics data of 1533 breast cancer specimens and identified an intriguing link among c-Myc, cancer stemness and copper-induced cell death (also known as “cuproptosis”). We screened out cuproptosis-related characteristics that predicts poor clinical outcomes and found that the pro-tumoral cuproptosis-based features were putatively enriched in MYC-targets and showed a significantly positive correlation with cancer stemness.

**Conclusion:**

In addition to previous reports on its oncogenic roles, c-Myc showed significant correlation to stemness phenotype and copper-induced cell toxicity in breast cancer tissues. Moreover, transcriptomics data demonstrated that pro-tumoral cuproptosis biomarkers had putative positive association with cancer stemness. This research combined clinical samples with large-scale bioinformatic analysis, covered description and deduction, bridged classic oncogenic mechanisms to innovative opportunities, and inspired the development of copper-based nanomaterials in targeting highly heterogeneous tumors.

**Supplementary Information:**

The online version contains supplementary material available at 10.1186/s12967-023-04204-5.

## Introduction

Breast cancer is the most common malignant tumor in women, leading to over 684,000 deaths according to latest report by CA Cancer J Clin [1, 2]. Though long-term prognosis of breast cancer is much better than other highly malignant tumors, such as melanoma, challenges including recurrence, distant metastasis and drug resistance still limit therapeutic effects and survival. Cancer stem cells (CSCs) are a self-renewal population critical to tumorigenesis, intra-tumoral heterogeneity, therapeutic resistance and recurrence [3]. Plasticity, quiescence, differentiative potential, and even its low mutational state means that CSCs have considerable research value [4–6].

High expression of CD44, CD133, ALDH and low expression of CD24 are well-acknowledged biomarkers in maintaining the stemness of CSC in solid tumors, including breast cancer and lung cancer [7–9]. For breast cancer cases, the CD44 high/CD24 low phenotype exacerbates aggressiveness, accelerates antitumor drug resistance and facilitates tumor progression [10, 11]. Moreover, even CSCs present varying phenotypes and spatial characteristics. Our previous research investigated clinical specimens of triple-negative breast cancer (TNBC), and termed this observation intra-tumoral stemness heterogeneity (ITSH) and concluded that ITSH is a promising biomarker of poor clinical outcomes [12]. CSCs differentiate into heterogeneous cancer cell clusters by asymmetrical division, decrease energy consumption in nutrition-deprived tumor microenvironment (TME), or maintain in a low-mutant state to survive immune attack [4]. However, molecular progenitors and driving mechanisms underlying cancer stemness are still elusive.

c-Myc elevation or hyperactivation exists in various cancers [13]. c-Myc exerts pro-tumoral effects via regulating cell cycle and activating downstream proliferative signaling pathways [14, 15]. In vitro experiments showed that c-Myc overexpression transforms normal breast epithelial cells into breast cancer cells and induces epithelial-mesenchymal transition (EMT) process [16]. For metastasis-initiating cells, cancer cell colonization is accompanied by increased proliferation and elevated c-Myc expression [17]. Clinically, c-Myc amplification is associated with greater benefit from trastuzumab for patients with HER2-positive breast cancer [18]. Our previous bioinformatic analysis proposed that c-Myc dysfunction is a potential regulator of cancer stemness and participates in rewiring intracellular molecular interactions and metabolisms [19, 20]. Therefore, in this research, we aimed to investigate the mechanisms between c-Myc, breast cancer stemness and clinical outcomes of patients.

Cuproptosis is a unique form of cell death induced by copper overloading that targets the tricarboxylic acid (TCA) cycle [21]. Some studies reported its role in cancer progression [22–24], but it remains to be seen whether this new concept offers druggable targets for use in clinical practice. In this study, we used transcriptomic profiling from large-scale databases of breast cancer patients, conducted rounds of screening and identified that cuproptosis was associated with poor clinical outcomes of breast cancer and cuproptosis engaged in c-Myc mediated breast cancer stemness. Thus, we proposed that cuproptosis-based risk scoring evaluation is conducive to predicting clinical outcomes of breast cancer. This research bridged the classic oncogene c-Myc and the new concept cuproptosis, proposed for the first time that cuproptosis may engaged in c-Myc-mediated breast cancer stemness, and identified a new cuproptosis-based scoring system that could be used for clinical applications.

## Materials and methods

### Clinical samples

Slices of breast cancer tissue and corresponding lymph nodes with cancer cell infiltration were collected from sixty patients after breast cancer surgery. Histological grading was performed based on the criteria of the World Health Organization (2019 World Health Organization classification of tumours of the breast) [25]. Pathologic staging was evaluated by pathologists according to the current International Union against Cancer Tumor Lymph Node Metastasis.

### IHC staining

IHC staining of breast tumor tissue samples was performed using antibodies against c-Myc, CD44, CD24, CD133 and ALDH1A1. Pathologic staging was determined by the current International Union against Cancer Tumor Lymph Node metastasis classification. Scanning files or images on paraffin-embedded specimens were collected by digital tissue scanner or imaging system, and the tissue measurement area was performed by pathologists. The number of weak, medium and strong positive cells in the measurement area was analyzed and calculated respectively (negative without staining, 0 points; weak positive light yellow, count 1 point; medium positive brown yellow, count 2 points; strong positive tan count 3 points), total cell count, positive cumulative optical density IOD value, positive pixel area, tissue area mm. The following results were calculated to reflect the degree of positivity [26].

### Data sources and normalization

Clinical information and gene expression profiling data (fragments per kilobase million, FPKM) of patients with Breast invasive carcinoma (BRCA) were downloaded from Gene Expression Omnibus (GEO) database and The Cancer Genome Atlas (TCGA) database. Three GEO BRCA cohorts (GSE42568, GSE48390, and GSE88770) and TCGA cohorts were obtained for subsequent analyses (Table 1) [27–30]. The FPKM values of TCGA-Breast invasive carcinoma (BRCA) were transformed into transcripts per kilobase million (TPM), as previously described, and were believed to be identical to those from microarrays. Four datasets were combined, and batch effect removal were performed by applying the “combat” algorithm of the SVA package, and the filtered expression matrix was normalized using Seurat’s NormalizeData function [31–34]. After data normalization, highly variable genes were identified and used for the following principal component analysis (PCA). A total of 1533 patients with BRCA from four data sets were enrolled in this study, including 104 patients from GSE42568, 81 patients from GSE48390, 117 patients from GSE88770, and 1091 patients from TCGA database [27–30]. The clinical variables included age, gender, grade, tumor stage, TNM Stage, HER2 mutation, ER mutation, follow-up time, and survival status.

**Table 1:** Clinical information of public datasets used in this study

Dataset	Size	Age	Tumor size	Pathology	Grade	Follow-up
GSE42568	104	31–89 years	0.6–8.0 cm	IDC: 82 ILC: 17 Other: 5	I: 11 II: 40 III: 53	Max: 3026 days Mean: 1887 days
GSE48390	81	< 70 years	NA	Invasive breast cancer	I: 5 II: 40 III: 36	0.1–5.8 years
GSE88770	117	35–89 years	0.9–1.7 cm	ILC	I–III	Min: 5 years

### Nonnegative matrix factorization (NMF) clustering analysis of cuproptosis biomarkers

Ten cuproptosis biomarkers (FDX1, LIAS, LIPT1, DLD, DLAT, PDHA1, PDHB, MTF1, GLS, CDKN2A) were retrieved from previous publications [21]. R package “NMF” was employed to divide all samples into two CuRGcluster 1 and CuRGcluster 2 according to expression of cuproptosis biomarkers [35]. This clustering was performed based on the following criteria. First, the cumulative distribution function (CDF) curve increased gradually and smoothly. Second, no groups had a small sample size. Lastly, after clustering, the intra-group correlation increased, while the inter-group correlation decreased.

### Relationship between molecular subtypes and the clinical features and prognosis of breast cancer

To examine the clinical value of the two subtypes identified by NMF clustering, we compared the relationships between molecular subtypes, clinicopathological characteristics, and prognosis. Clinicopathological features included age, gender, grade, tumor stage, TNM stage, HER2 mutation, ER status, follow-up time, and survival status. Furthermore, the differences in overall survival (OS) among different subtypes were assessed using Kaplan–Meier curves generated by R packages “survival” and “survminer” [36].

### Correlations of cuproptosis-based clustering with TME in breast cancer

The ESTIMATE algorithm was employed to evaluate the immune and stromal scores of each patient [37]. In addition, the proportion of 22 human immune cell subsets of every breast cancer sample were calculated by the CIBERSORT algorithm [38]. Furthermore, immune cell infiltration in the breast cancer TME were also determined using a single-sample gene set enrichment analysis (ssGSEA) algorithm [39].

### Differentiated expressed genes (DEGs) identification and functional annotation

DEGs between CuRGcluster C1 and C2 were identified using R package “limma” with a fold-change of 0.05 and an adjusted p-value of < 0.05. In order to further explore the potential functions of cuproptosis pattern-related DEGs and identify the related gene functions and enriched pathways, functional enrichment analyses were executed on the DEGs using the R package “clusterprofiler”.

### Construction of the cuproptosis-related prognostic CuRG_score

To investigate whether cuproptosis was associated with the outcomes of breast cancer patients, we aimed to establish a CuRG_score. First, the top three highly expressed cuproptosis-related genes in geneclusterC were exposed to the univariate Cox regression analysis and two significant genes were subsequently exposed to a LASSO cox regression analysis to compute the exact coefficient values of each identified association. Then, all BRCA patients were randomly categorized into training (n = 686) and testing (n = 685) sets at a ratio of 1:1, then the former was used to construct the cuproptosis-related prognostic CuRG_score. Eventually, two candidate genes were established to get a prognostic CuRG_score in the training set. The CuRG_score was calculated as: CuRG_score = -0.469160009286577*(LIPT1) + 0.322953604620369*(DLAT). Based on the median risk score, a total of 686 patients in the training set were divided into low-risk (CuRG_score < median value) and high-risk (CuRG_score > median value) groups and then subjected to Kaplan–Meier survival analysis. Afterwards, principal component analysis (PCA) was performed using R package “ggplot2”. Similarly, the testing and all sets were divided into low- and high-risk groups, each of which was subjected to Kaplan–Meier survival analysis and the generation of receiver operating characteristic (ROC) curves.

Clinical correlation and stratification analyses of the CuRG_score. Chi-square tests were used to explore the relationships between the CuRG_score and the clinical characteristics (age, gender, TNM stage, HER2 status, and ER status). To assess whether risk scores were independent of other available clinicopathological features, we subjected the training and testing sets to univariate and multivariate analyses. In addition, we performed a stratified analysis to determine whether the CuRG_score retained its predictive ability in different subgroups according to age, gender, T stage, N stage, M stage, tumor stage, HER2 status, and ER status.

### Evaluation of immune status, microsatellite instability (MSI), and stemness index between the high- and low-risk groups

To evaluate the proportions of tumor infiltrating immune cells in the TME, CIBERSORT was employed to quantify the abundance of 22 infiltrating immune cells in heterogeneous samples in the low- and high-risk groups. We explored the associations between the fractions of 22 types of infiltrating immune cells and eight genes in the CuRG_score. Boxplots were used to examine the differential expression levels of immune checkpoints between the low- and high-score groups. Furthermore, we analyzed the relationships between the two risk groups and MSI and CSC.

### Mutation and drug susceptibility analysis

To determine the somatic mutations of breast cancer patients between high- and low-risk groups, the mutation annotation format from the TCGA database was generated using the “maftools” R package. We also calculated the tumor mutation burden (TMB) score for each patient with BRCA in the two groups. To explore differences in the therapeutic effects of chemotherapeutic drugs in patients in the two groups, we calculated the semi-inhibitory concentration (IC50) values of chemotherapeutic drugs commonly used to treat BRCA using the “pRRophetic” package.

### Establishment and validation of a nomogram scoring system

The clinical characteristics and risk score were used to develop a predictive nomogram using the “rms” package based on the outcome of the independent prognosis analysis. In the nomogram scoring system, each variable was matched with a score, and the total score was obtained by adding the scores across all variables of each sample. Time-dependent ROC curves for 1-, 3-, and 5-year survivals were used to assess the nomogram. Calibration plots of the nomogram were used to depict the predictive value between the predicted 1-, 3-, and 5-year survival events and the virtually observed outcomes.

### Statistical analyses

All statistical analyses were performed using R version 4.1.0. Statistical significance was set at p < 0.05.

## Results

### c-Myc was a potential regulator of breast cancer intra-tumoral heterogeneity

**Fig. 1:**
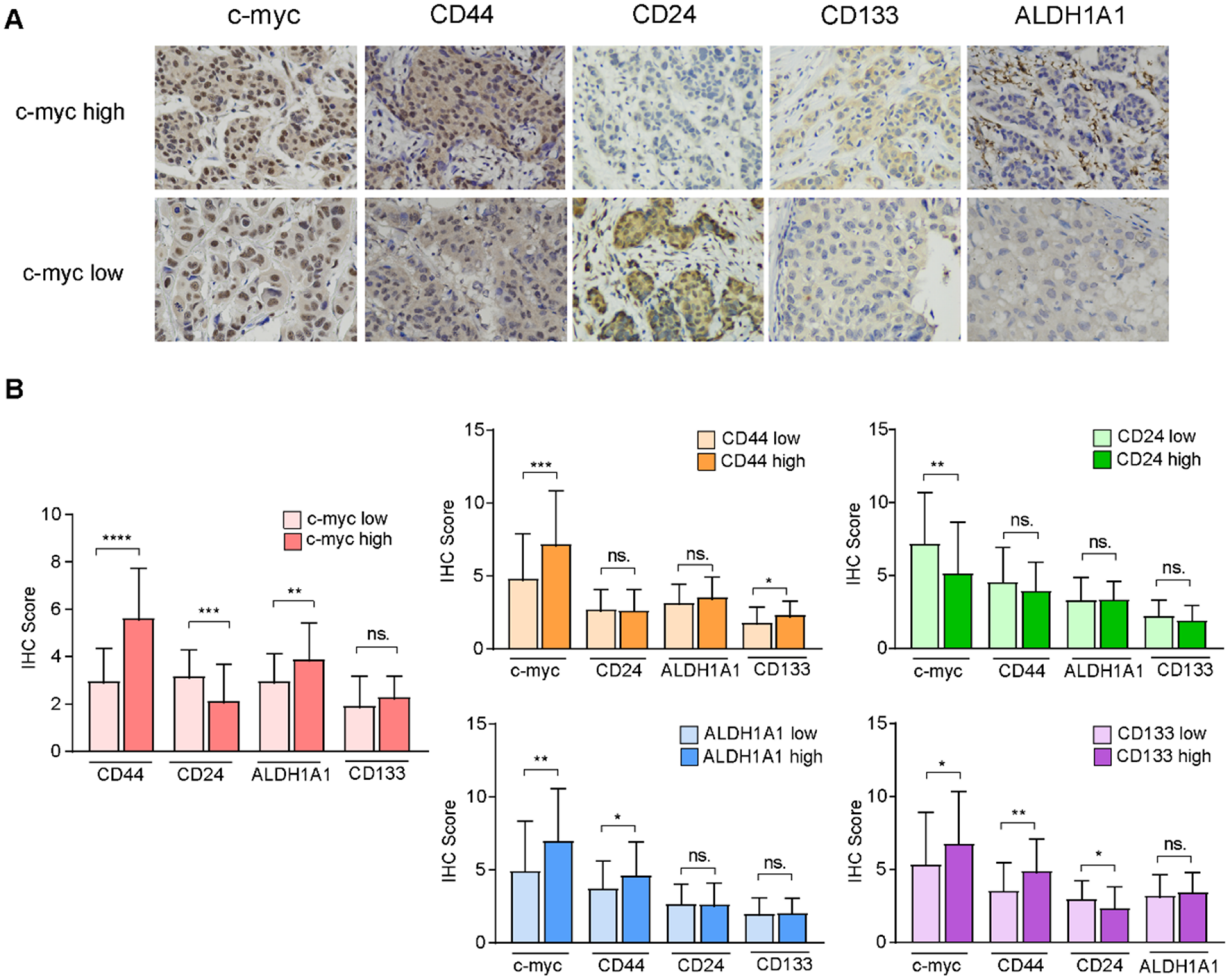
Immunohistochemical staining demonstrates the positive association between c-Myc and BCSC phenotype. **A** Pathological slides staining BCSC biomarkers (CD44, CD24, CD133 and ALDH1A1) in c-Myc-high and c-Myc-low group. **B** Statistical analysis scoring expression of c-Myc and BCSC biomarkers demonstrating that in c-Myc high group, BCSC biomarkers have higher expression. (BCSC, breast cancer cell.) (*P < 0.05; **P < 0.01; ***P < 0.001; ****P < 0.0001)

We first investigated the expression of the oncoprotein c-Myc and four breast cancer stem cell (BCSC) biomarkers: CD44, CD24, CD133 and ALDH1A1 [40]. We performed immunohistochemical (IHC) staining on surgical specimens of the primary cancer tissue (PC) and an axillary lymph node with cancer evasion (LN +). Paired surgical specimens were derived from 60 patients diagnosed with various breast cancer subtypes (Table S1). For 60 breast cancer cases, paraffin-embedded tissue from primary cancers and lymph nodes was available for a total of 120 samples. Protein expression of each sample was assessed based on mean expression of three slices. Then, we evaluated the expression of c-Myc, CD44, CD24, CD133, and ALDH1A1 of sixty primary cancers and paired lymph nodes with cancer evasion (Fig. 1A). In both PC and LN + , c-Myc expression positively correlated with CD44, CD133, and ALDH1A1, and negatively correlated with CD24 (Fig. 1B and Table 2). This finding verified the positive correlation between c-Myc and BCSC biomarkers [41–44]. Since these tissues were collected from paired PC and LN + of the same patients, we also performed Spearman’s correlation analysis on stemness biomarkers in respective tumor sites and identified that a more prominent positive correlation between c-Myc and BCSC phenotype in PC compared with LN + (Table 2), suggesting a potentially more prominent regulation of c-Myc to stemness in the primary tissue. This finding was consistent with our previous report that PC-derived BCSC differentiated into cancer cell subclusters with better metastatic potential [4]. Therefore, based on IHC staining of 120 breast cancer surgical specimens, we identified the positive correlation between c-Myc and BCSC phenotypes, and supposed that the oncoprotein c-Myc was a driving regulator of cancer stemness in breast cancer.

**Table 2:** Spearman’s rank correlation between c-Myc, CD44, CD24, CD133 and ALDH1A1 in primary cancer and positive lymph nodes of our breast cancer cohort

*R*_PC_ (P value)	C-myc	CD44	CD24	ALDH1A1	CD133
C-myc		0.543** (0.000)	− 0.460** (0.000)	0.356** (0.006)	0.213 (0.105)
CD44	0.543** (0.000)		− 0.082 (0.536)	0.184 (0.166)	0.176 (0.182)
CD24	− 0.460** (0.000)	− 0.082 (0.536)		− 0.115 (0.389)	− 0.195 (0.139)
ALDH1A1	0.356** (0.006)	0.184 (0.166)	−0.115 (0.389)		0.239 (0.068)
CD133	0.213 (0.105)	0.176 (0.182)	−0.195 (0.139)	0.239 (0.068)	
*R*_LN_ + (P value)					
C-myc		0.428** (0.001)	− 0.234 (0.075)	0.312* (0.017)	0.043 (0.747)
CD44	0.428** (0.001)		− 0.119 (0.369)	0.153 (0.253)	0.253 (0.053)
CD24	− 0.234 (0.075)	− 0.119 (0.369)		0.047 (0.729)	− 0.117 (0.376)
ALDH1A1	0.312* (0.017)	0.153 (0.253)	0.047 (0.729)		− 0.117 (0.382)
CD133	0.043 (0.747)	0.253 (0.053)	− 0.117 (0.376)	− 0.117 (0.382)	

(*P < 0.05; **P < 0.01.)

### Cuproptosis was dysregulated in breast cancer tissues

**Fig. 2:**
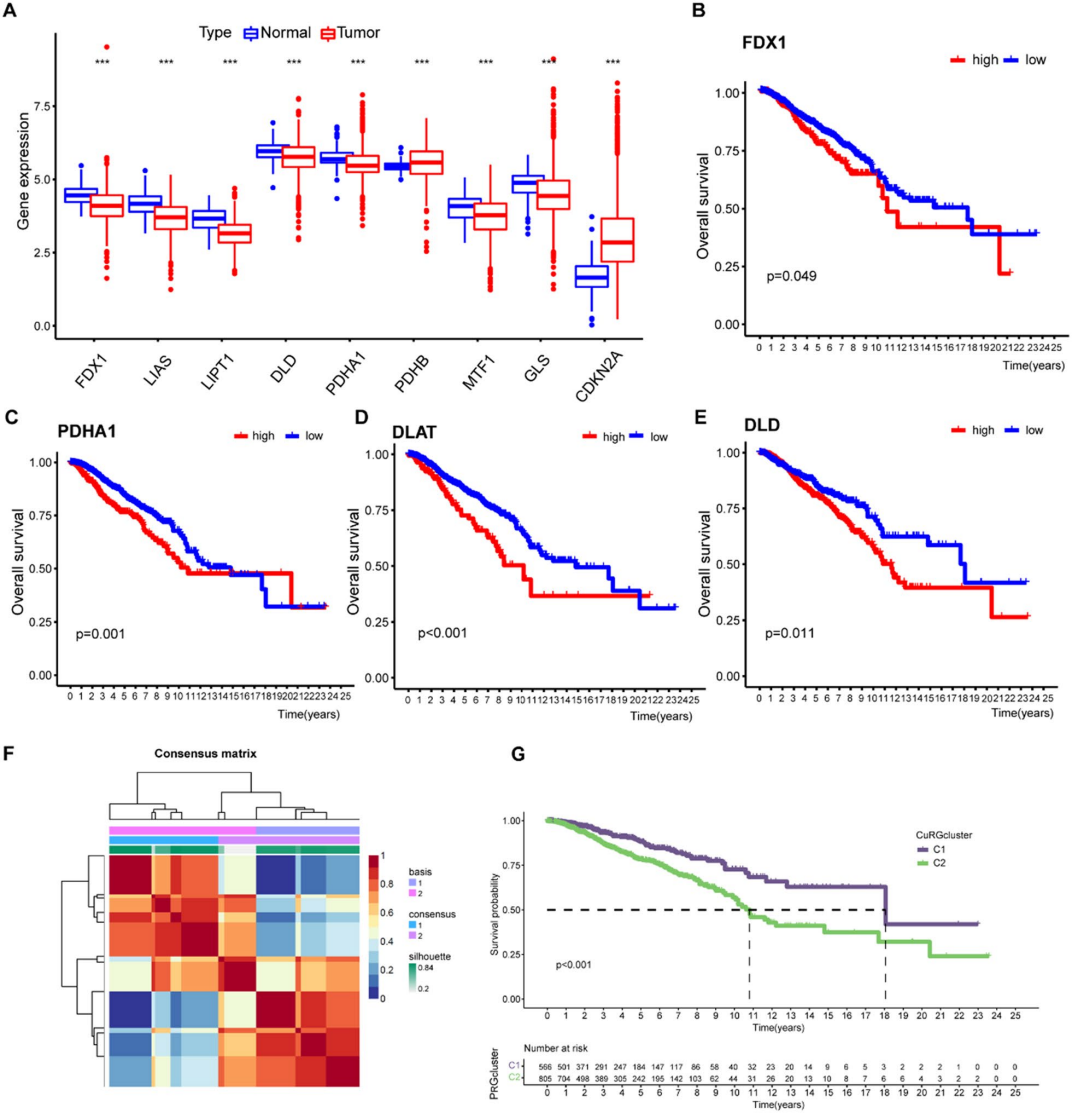
Primary clustering of breast cancer patients by cuproptosis-related genes. **A** Expression of cuproptosis-related genes in breast cancer tissues and normal control. **B**–**E** Representatives of Kaplan–Meier plots showing the relationship between cuproptosis-related genes and overall survival. **F** Consensus matrix clustered all breast cancer patients into two clusters based on the cuproptosis-related genes. **G** Survival curves of patients from two CuRGclusters. (*P < 0.05; **P < 0.01; ***P < 0.001)

Copper-induced cell death (cuproptosis) is a newly reported form of cell death. Excessive copper binds to constituents of the TCA cycle in mitochondria, leading to lipoylated protein aggregation, proteotoxic stress and ultimately cell death [21]. Seven genes (FDX1, LIAS, LIPT1, DLD, DLAT, PDHA1, and PDHB) were verified to rescue cells from copper toxicity, and three genes (MTF1, GLS, and CDKN2A) exacerbated cell death induced by copper overloading. Since dysregulation of intracellular homeostasis and cell cycle contribute to multiple pathologies including cancer [45], we interrogated the expression of cuproptosis biomarkers in breast cancer specimens (Fig. 2A). We conducted transcriptome analysis on a large-scale sample derived from four open databases (TCGA, GSE42568, GSE48390, and GSE88770). Nine out of ten cuproptosis biomarkers were differentially expressed between breast cancer tissues and normal control. PDHB and CDKN2A expression were significantly higher in breast cancer tissues compared with control. PDHB encodes pyruvate dehydrogenase, which links glycolysis to the TCA cycle and can be mediated by the oncogenic AMPK signaling pathway [46]. Moreover, murine models demonstrated that ectopic activation of the pyruvate dehydrogenase complex by exogenous expression of PDHB increased metastatic potential and survival of cancer cells [47]. CDKN2A is a tumor suppressor gene and its target, p16, inactivates the cyclinD-CDK4/6 complex and leads to cell cycle arrest [48]. CDKN2A alteration is a contributor of tumorigenesis, however, its overexpression was also reported in several types of cancers due to its association with aberrant apoptosis, senescence, angiogenesis and cancer migration [49, 50]. Inverse correlation between Rb and p16 was reported in several cancers, including breast cancer. Additionally, p16 overexpression and heterozygous Rb loss are predictors to CDK4/6 resistance in hormone receptor-positive breast cancer [51]. Survival analysis demonstrated that among the cuproptosis biomarkers, FDX1, PDHA1, DLAT, and DLD predicts poor prognosis (Fig. 2B–E).

### Identifying predictive biomarkers of breast cancer based on cuproptosis

**Fig. 3:**
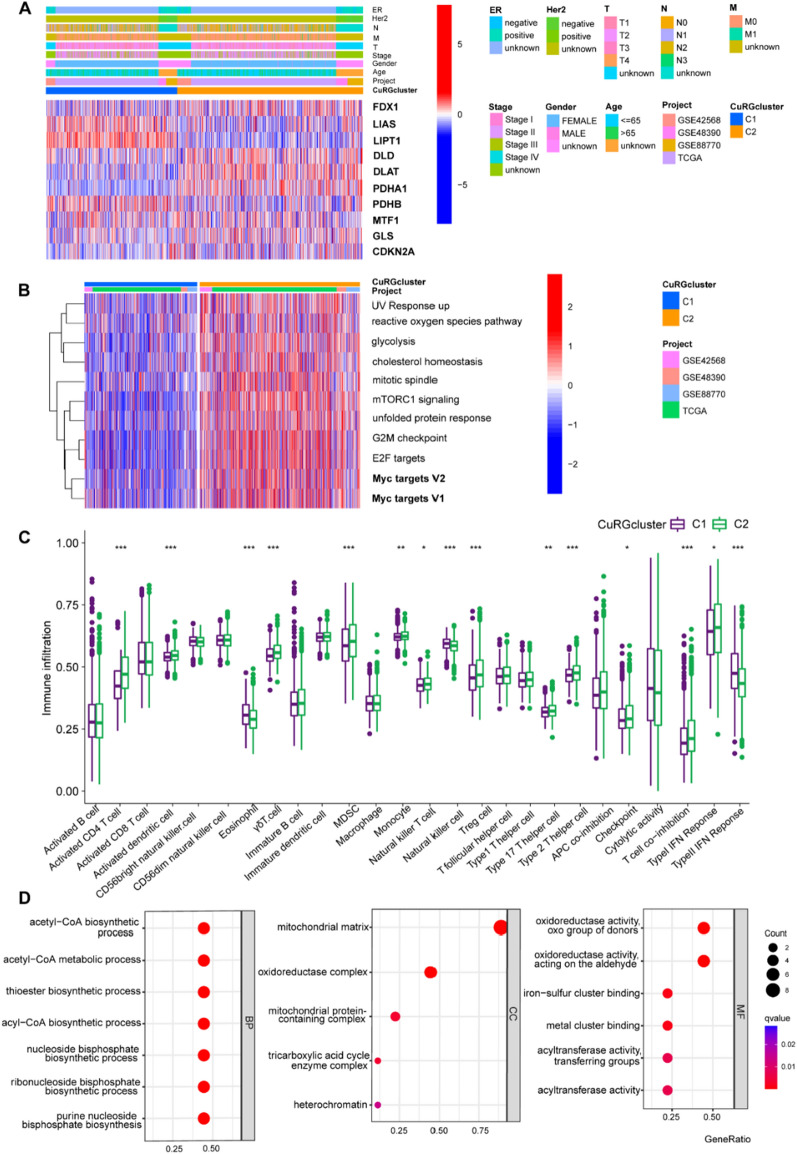
Cuproptosis-related poor prognosis is a potential target of c-Myc. **A** Heatmap showing cuproptosis-related genes of breast cancer patients. The columns above illustrate the clinicopathological features of patients. **B** Top pathways enriched by cuproptosis-related genes that mediated poor breast cancer prognosis. **C** Immune components identified by bioinformatic analysis of two clusters. **D** Top pathways enriched by two CuRGclusters according to transcriptomic profiling. (BP, biological process; CC, cellular component; MF, molecular function.) (*P < 0.05; **P < 0.01; ***P < 0.001)

To further identify the potential correlation between cuproptosis and breast cancer prognosis, we conducted unsupervised clustering on all breast cancer cases and grouped all patients into two CuRGClusters (C1 and C2, Fig. 2F). Bioinformatic analysis of the survival curve for the two CuRGClusters identified that patients from C2 had worse prognosis than C1 (Fig. 2G). We then investigated the expression of cuproptosis-related genes in the two CuRGClusters. Expression of FDX1, DLD, DLAT, PDHA1, GLS and CDKN2A were more enriched in the cluster with worse prognosis (Fig. 3A). C1 showed significantly higher expression of LIAS, LIPT1, and PDHB. C2 showed significantly higher expression of FDX1, DLD, DLAT, PDHA1, GLS, and CDKN2A. For C2, we identified increased enrichment of pathways including ROS pathway, glycolysis and mTORC1 signaling. Because cuproptosis-mediated cellular damage mainly targets mitochondrial TCA, and c-Myc was reported to maintain breast cancer stemness by increasing oxidative phosphorylation (OXPHOS) and producing reactive oxygen species (ROS) [52], we interrogated the enrichment of c-Myc-associated pathways in these breast cancer specimens. Notably, we found that tissue samples of C2 cluster showed putative enrichment of c-Myc-targeted pathways (Fig. 3B). This finding prompted us to speculate that cuproptosis was the potential downstream of c-Myc-mediated tumor progression. Additional file 1.

To investigate whether cuproptosis exerts effects on the TME, we evaluated the immune cell infiltration of breast cancer patients. In the breast cancer subgroup with worse prognosis, inhibitory immune response was identified by a higher proportion of inhibitory T cells and immunosuppressive components represented by myeloid-derived suppressor cells (MDSC) and regulatory T (Treg) cells (Fig. 3C). A larger proportion of γδT also suggested immune tolerance in the breast cancer samples from C2. γδT is a CD4-negative and CD8-negative T-cell subgroup having both anti- and pro-tumoral functions during cancer progression [53]. On the one hand, γδT present antigens and exert direct cytotoxicity, on the other, certain γδT subgroups express exhausted ligands, facilitate pro-tumoral chronic inflammation and attenuate adaptive immunity [54]. Based on transcriptomics and the enriched functional pathways, both CuRGClusters (C1 and C2) are active in mitochondrial biological functions such as acetyl-CoA metabolism, purine nucleoside bisphosphate biosynthesis, and reducing equivalents production (Fig. 3D).

### Cuproptosis was predictive to clinical outcomes of breast cancer

**Fig. 4:**
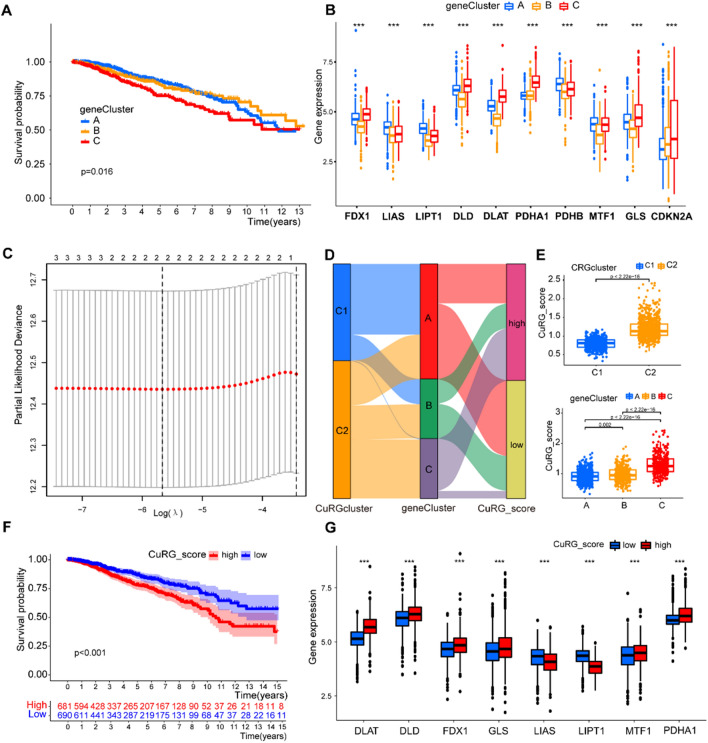
Key gene panel that mediates cuproptosis-related poor prognosis of breast cancer samples. **A**, **B** Secondary clustering based on significantly expressed cuproptosis-related genes grouped breast cancer patients into three geneClusters with different prognosis. **C** Nomogram performed in preparation for CuRG_score establishment. **D**, **E** The corresponding relationship of previous clustering. **F** Survival curves of two subclusters divided by the CuRG_score. **G** Expression of cuproptosis-related genes in the high- and low-CuRG_score groups. (*P < 0.05; **P < 0.01; ***P < 0.001)

To further identify the pro-tumoral effects of cuproptosis-related mechanisms and compute the exact coefficient values of each identified association, we performed univariate Cox regression analysis on the differentially expressed cuproptosis biomarkers of C1 and C2. The univariate Cox regression demonstrated that LIPT1, PDHA1, and DLAT as predictor to poor survival and further classified breast cancer samples into three geneClusters (geneClusterA, geneClusterB and geneClusterC, Fig. 4A, B). Then, we performed LASSO regression analysis on the three genes to establish a CuRG_score that evaluate the degree of cuproptosis in breast cancer samples (Fig. 4C). Eventually, we got the CuRG_score calculated as: CuRG_score =  − 0.469160009286577* (LIPT1) + 0.322953604620369* (DLAT). Breast cancer samples were divided into the high- and low-risk group based on CuRG_score (Fig. 4D). We checked the correspondence between CuRGclusters classified by differentially expressed cuproptosis biomarkers (C1 and C2), geneClusters classified by univariate Cox regression (A, B, C), and the risk groups classified by CuRG_scores (Fig. 4E). It turned out that previous clusters with comparatively poor outcomes (C2 compared with C1, geneClusterC compared with geneClusterA and geneClusterB) showed putatively higher scores of cuproptosis. Furthermore, survival curve further verified that higher CuRG_score is associated with poorer clinical prognosis (Fig. 4F). The high-risk group had higher expression of DLAT, DLD, FDX1, GLS, MTF1, and PDHA1, and lower expression of LIAS and LIPT1 compared with the low-risk group (Fig. 4G). Based on the above analysis of large-scale transcriptomics, we identified that cuproptosis was predictive to poorer clinical outcomes of patients with breast cancer.

### Cuproptosis engaged in c-Myc-mediated cancer stemness

**Fig. 5:**
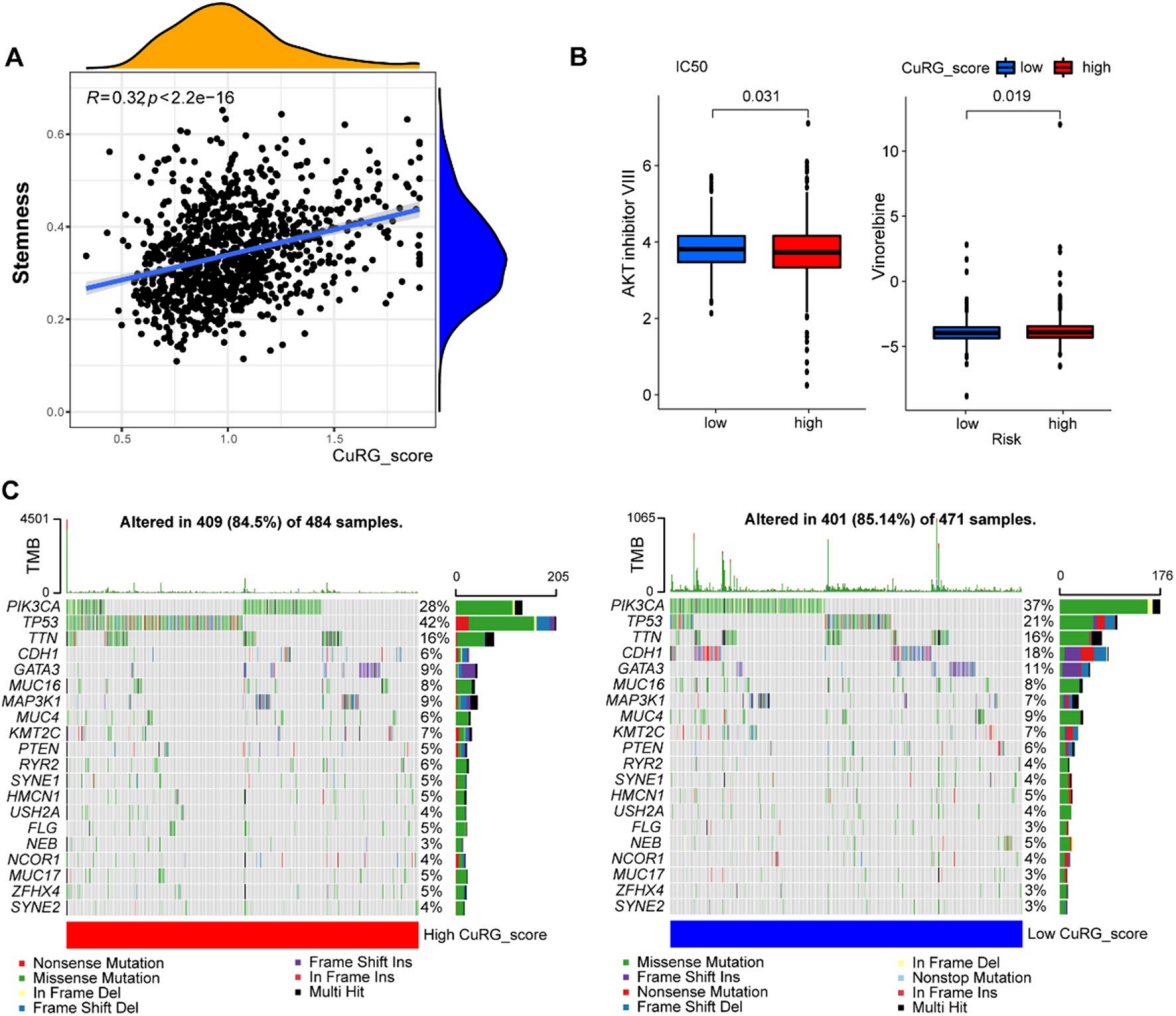
Characteristics of cuproptosis-predicted breast cancer samples. **A** Bioinformatic analysis showed positive correlation between CuRG_score and cancer stemness. **B** Drug sensitivity of AKT inhibitor and vinorelbine in two groups with high and low CuRG_score. **C** TMB of classic oncogenes showed that the high-risk group had a lower TMB compared with the low-risk group. (TMB, tumor mutation burden.)

c-Myc is an oncogene regulating cancer cell proliferation through multiple mechanisms and modulating tissue-specific chemotherapy sensitivity via mitochondrial apoptosis [55]. Moreover, c-Myc contributes to maintaining BCSC by increasing OXPHOS and producing ROS [52]. The above bioinformatic analysis identified that cuproptosis predicted poor clinical outcomes of patients with breast cancer, and suggested that this pro-tumoral effect was significantly associated with c-Myc-mediated downstream targets, we inferred that cuproptosis engaged in c-Myc-mediated tumor progression. To further investigate whether the relationship between c-Myc and cuproptosis was also associated to cancer stemness, an embodiment of c-Myc-mediated carcinogenic property, we performed another correlation analysis on stemness and cuproptosis. We found a significant positive correlation between CuRG_score and breast cancer stemness phenotype (Fig. 5A). These findings collectively indicated that cuproptosis [21] was engaged in the c-Myc-mediated breast cancer stemness and malignancy.

It is acknowledged that cancer stem cell is a regulator of intra-tumoral heterogeneity and a marker of chemo- and radio-resistance. To test whether this CuRG_score predicted therapeutic effects, we investigated the sensitivity to several anti-tumor drugs that were frequently used. The high-risk group had better responses to AKT inhibitors and vinorelbine than the low-risk group (Fig. 5B). AKT inhibitors in combination with first-line chemotherapy [56] and vinorelbine-based combinatorial therapy [57, 58] both showed therapeutic effects in breast cancer cases, and we demonstrated that high CuRG_score was significantly associated with higher IC_50_ of these drugs. TMB has promising predictive ability to identify patients who derive clinically significant improvement in survival from immunotherapy [59, 60]. Bioinformatic analysis demonstrated that high CuRG_score was correlated with lower TMB (Fig. 5C). These findings suggested that cuproptosis engaged in c-Myc-mediated breast cancer stemness, therefore rendering breast cancer cells in a dormient and less mutative state that is insensitive to chemotherapy and immunotherapy.

## Discussion

Since the first report of copper-induced cell death in March 2022, there has been a great deal of interest in this novel concept. So far, various bioinformatic analysis have linked this new form of cell death to multiple diseases, including cancers and non-malignant diseases such as rheumatoid arthritis [61, 62]. Moreover, revelation of the exact mechanisms also boosted copper-based nanomaterials and enriched optional carrier for cancer treatment [62]. Though various studies have established prognostic models or simulated the tumor immune microenvironment based on public database, few focused on the molecular mechanisms and the therapeutic potential of this newly identified cell death. The Science report verified ten genes related to cuproptosis among which seven genes (LIAS, LIPT1, DLD, DLAT, PDHA1, PDHB, FDX1) rescued cells from copper overloading and mitochondrial failure, while the other three (MTF1, GLS, CDKN2A) offers synergy and promotes cuproptosis [21]. Another form of metal-dependent cell death that arouses enormous interest is ferroptosis. After ferroptosis was reported, researchers expanded their study into the molecular pathways and utilized cell death resulted from ferroptosis-mediated lipid peroxide accumulation to target cancerous proliferation. The key enzymes and regulatory mechanisms involved in cuproptosis have been identified. However, its role in pathological conditions as well as the potential in clinical practice remains unclear. Inspired by ferroptosis-mediated cancer biology, researchers devoted to the potential value of cuproptosis in cancer. By combining copper toxicity with RNA methylation, a report on hepatocellular carcinoma demonstrates the RNA regulators of cuproptosis biomarkers and shows correlation with tumor mutation which partly predicts sensitivity to immunotherapy [63]. Moreover, studies in combination with lncRNA network indicate that cuproptosis-related lncRNAs can predict the recruitment and infiltration of immune components in TME [64]. These interesting findings motivate us to take cuproptosis as an opportunity to extend our research in oncology.

Heterogeneity is one of the challenges of cancer treatment. Intratumoral heterogeneity, demonstrated in spatiotemporal evolution and instability, greatly contributed to failure of monotherapy, therefore leading to tumor progression and metastasis. CSCs are a unique cancer cell population comparatively immune-privileged than other subclusters that drive the initiation, metastasis and therapeutic resistance of most neoplasms [65, 66]. Investigation into heterogeneity, as well as the contributing factors underlying this intratumoral heterogeneity, is highly valuable to research and clinical practice. c-Myc has been recognized as a “grand orchestrator” of carcinogenesis that mediates tumor progression. In addition to being an important transcriptional factor of cancer cells, c-Myc dysregulates the TME and remodels antitumor immunity [67].

Our previous bioinformatic analysis suggested that c-Myc was a driving factor of breast cancer stemness. Therefore, we aimed to further specify the specific mechanisms by which c-Myc mediates breast cancer stemness and intratumoral heterogeneity. Start with IHC staining of breast cancer surgical specimens, we verified that c-Myc is putatively related to BCSC phenotype. Our analysis based on large-scale transcriptomics profiles supported that cuproptosis-related poor prognosis is a downstream target of c-Myc-mediated mechanisms. Further statistical analysis and screening established a CuRG_score as an indicator to poor prognosis and showed putative correlation with breast cancer stemness. The above findings proposed a novel mechanism of cuproptosis in breast cancer, that is cuproptosis engaged in breast cancer stemness via c-Myc-related mechanisms. We proposed that cuproptosis was a potential target of the classic oncogene c-Myc, the intermediator of c-Myc-mediated breast cancer stemness and a predictor to poor prognosis of breast cancer.

## Conclusion

In conclusion, based on a sample size of 1,533 breast cancer cases, we combined large-scale transcriptomics analysis and pathological examination and revealed that cuproptosis engaged in c-Myc-mediated breast cancer stemness. This finding suggests the potential of innovative nanomaterials that pack c-Myc-targeted inhibitors with copper polymers.

## Supplementary Information

**Additional file 1. Table S1 **Clinicopathological features of breast cancer samples for IHC staining.

## Data Availability

Data used for bioinformatic analysis in this manuscript was acquired from the GEO database (index number: GSE42568, GSE48390, and GSE88770).
